# Women’s views on accepting COVID-19 vaccination during and after pregnancy, and for their babies: a multi-methods study in the UK

**DOI:** 10.1186/s12884-021-04321-3

**Published:** 2022-01-14

**Authors:** Helen Skirrow, Sara Barnett, Sadie Bell, Lucia Riaposova, Sandra Mounier-Jack, Beate Kampmann, Beth Holder

**Affiliations:** 1 Department of Primary Care and Public Health, School of Public Health, Imperial College London, London, UK; 2 Department of Metabolism, Digestion and Reproduction, Institute of Reproductive and Developmental Biology, Imperial College London, London, UK; 3 Department of Global Health and Development, Faculty of Public Health and Policy, London School of Hygiene & Tropical Medicine, London, UK; 4 The Vaccine Centre, Faculty of Infectious and Tropical Diseases, London School of Hygiene & Tropical Medicine, London, UK; 5 Vaccines and Immunity Theme, MRC Unit The Gambia at London School of Hygiene and Tropical Medicine, Banjul, The Gambia

## Abstract

**Background:**

COVID-19 vaccines are advised for pregnant women in the United Kingdom (UK) however COVID-19 vaccine uptake among pregnant women is inadequate.

**Methods:**

An online survey and semi-structured interviews were used to investigate pregnant women’s views on COVID-19 vaccine acceptability for themselves when pregnant, not pregnant and for their babies. One thousand one hundred eighty-one women, aged over 16 years, who had been pregnant since 23rd March 2020, were surveyed between 3rd August–11th October 2020. Ten women were interviewed.

**Results:**

The majority of women surveyed (81.2%) reported that they would ‘definitely’ or were ‘leaning towards’ accepting a COVID-19 vaccine when not pregnant. COVID-19 vaccine acceptance was significantly lower during pregnancy (62.1%, *p* < 0.005) and for their babies (69.9%, p < 0.005). Ethnic minority women were twice as likely to reject a COVID-19 vaccine for themselves when not pregnant, pregnant and for their babies compared to women from White ethnic groups (*p* < 0.005). Women from lower-income households, aged under 25-years, and from some geographic regions were more likely to reject a COVID-19 vaccine when not pregnant, pregnant and for their babies. Multivariate analysis revealed that income and ethnicity were the main drivers of the observed age and regional differences. Women unvaccinated against pertussis in pregnancy were over four times more likely to reject COVID-19 vaccines when not pregnant, pregnant and for their babies. Thematic analysis of the survey freetext responses and interviews found safety concerns about COVID-19 vaccines were common though wider mistrust in vaccines was also expressed. Trust in vaccines and the health system were also reasons women gave for accepting COVID-19 vaccines.

**Conclusion:**

Safety information on COVID-19 vaccines must be clearly communicated to pregnant women to provide reassurance and facilitate informed pregnancy vaccine decisions. Targeted interventions to promote COVID-19 vaccine uptake among ethnic minority and lower-income women may be needed.

**Supplementary Information:**

The online version contains supplementary material available at 10.1186/s12884-021-04321-3.

## Background

On the 16th April 2021, the United Kingdom’s (UK) Joint Committee on Vaccination and Immunisation (JCVI) announced that pregnant women should be offered the COVID-19 vaccine ‘*at the same time as the rest of the population, based on their age and clinical risk group’*. [1] Given this guidance, understanding pregnant women’s’ perspectives on the acceptability of being vaccinated against COVID-19 is vital. We present here the first multi-methods study exploring UK women’s views on the acceptability of COVID-19 vaccination in pregnancy, as well as their views on vaccination for their babies, and for themselves when not pregnant.

At the start of the SARS-CoV-2 pandemic in 2020, there was a lack of evidence on the risk of COVID-19 disease in pregnant women [2]. It is now known that while pregnant women do not appear to be at greater risk of contracting SARS-CoV-2 there is a small risk of severe illness with COVID-19 disease, particularly in the last trimester of pregnancy (i.e. from 28 to 40 weeks of pregnancy) [2–4]. Since the beginning of the pandemic in the United Kingdom (UK), as a precaution pregnant women have been classed as ‘vulnerable’ to COVID-19 and advised to carefully adhere to social distancing guidance by the National Health Service (NHS), particularly in the third trimester of pregnancy [5, 6].

Despite calls by experts [7], pregnant women were not included in the initial COVID-19 vaccine trials, though COVID-19 vaccine trials involving pregnant women have now started [8]. In the UK, the general COVID-19 vaccination programme began in December 2020 with prioritisation of those at greater risk of hospitalisation or being severely ill with COVID-19 and those caring for vulnerable individuals, such as health and social care workers [9]. Initial guidance from the UK’s Joint Committee on Vaccination and Immunisation (JCVI), was that pregnant women should not be offered COVID-19 vaccination due to a lack of data on the safety of COVID-19 vaccines during pregnancy [9]. In contrast, in the United States of America (USA) the Centers for Disease Control and Prevention (CDC) advised that pregnant women could be offered COVID-19 vaccination with information available to enable pregnant women to make informed decisions [10]. The UK guidance was changed on December 30th 2020 [11], with pregnant women at greater risk of contracting COVID-19 (e.g. frontline healthcare workers) or at greater risk of severe disease due to other risk factors being able to be vaccinated following a discussion with a healthcare professional [11]. Given the availability of a larger databases on vaccine safety following the introduction of the vaccines, primarily from the USA [12], this recommendation was then amended to include all pregnant women in line with the rest of the population, with mRNA vaccines identified as the preferred product to be offered [1].

Ethnic minorities are at higher risk of dying from COVID-19 [13], and in the UK pregnant women from Black or other ethnic minority groups are overrepresented among women admitted to hospital with COVID-19 infection during pregnancy [4]. Work by Bell et al. found that parents from ethnic minority backgrounds other than White in the UK are less likely to accept a COVID-19 vaccine for their children [14]. This finding is consistent with other reports that individuals from ethnic minorities are less likely to accept COVID-19 vaccination for themselves [15, 16]. For example, the Office for National statistics in the UK found that 21% of Black or Black British adults have either declined or are unlikely to accept COVID-19 vaccination compared to only 4% of White adults [17]. We have previously shown that acceptance of pertussis and influenza vaccines in pregnancy is also lower in this group [18, 19].

Parental decisions about childhood vaccinations have also been shown to begin in pregnancy [20], so it is therefore useful to assess pregnant women’s perspectives on COVID-19 vaccines for both themselves and their children. Understanding women’s views and acceptability of COVID-19 vaccination is also important to address given that over 98% of pregnant women admitted to hospital with COVID-19 between 1st February 2021 to 30th September 2021 were unvaccinated [21].

We conducted a multi-methods study to investigate the views of pregnant women in the UK on the likely uptake of a future COVID-19 vaccine for themselves and their children. At the time of the survey no COVID-19 vaccines had been licensed for use but there was a recognition that COVID-19 vaccination could be made routinely available to pregnant women, children, and women of childbearing age in the future.

## Methods

A multi-methods approach was taken – using quantitative and qualitative components – with the aim of gaining insight into the acceptability of future COVID-19 vaccines for pregnant women and their children. The data presented here is part of a larger survey aimed at investigating the impact of the COVID-19 pandemic on access, awareness, and acceptance of routine maternal vaccines. The study comprised of a questionnaire survey and semi-structured interviews, to both quantify different views on accepting COVID-19 vaccines and then to also explore views in more depth.

### Survey recruitment

Eligible participants were required to have been pregnant at some point between the start of the UK 2020 lockdown (from 23rd March 2020) and the time of survey completion, to be resident in the UK, and to be aged 16 years or over. The survey was live from 3rd August – 11th October 2020. The online survey was prefaced by an information page explaining the study, and how the data was to be used. Participants were informed that by taking part in the survey, they agreed for their responses to be used for research purposes. Participants were required to confirm (by tick-box) at the start of the survey that they met the eligibility criteria and that they consented to participate.

The survey was advertised and promoted using Facebook with a Facebook landing page and paid advertising using Facebook’s ad manager which cross posts to Instagram. The three adverts had a combined reach of 46,146, 1573 post engagements and 1394 link clicks. Related organisations on Facebook were also contacted individually by study researchers, including pregnancy yoga and birth preparation classes, breastfeeding support groups and toddler groups. The survey was shared and distributed via the research team’s personal twitter accounts including linking to other researchers and organisations with maternal and vaccine uptake interests. Finally, the survey was also promoted via some Maternity Voices Partnerships [22] who were e-mailed and invited to share the survey, and via a post on the website Mumsnet.

### Survey design

The survey was designed with input from midwives, pregnancy vaccine researchers, paediatricians and public health professionals and was based on previous research surveys on pregnancy vaccination [18] and other surveys that had been used to assess COVID-19 vaccine views during the pandemic [14]. Here we present one aspect of the survey, specifically regarding the acceptability of a ‘future’ COVID-19 vaccination. The COVID-19 vaccine section asked: ‘Please select how much you agree or disagree with the following statements about a future vaccine to protect against COVID-19: i) If a vaccine against coronavirus (COVID-19) becomes available, I would get vaccinated whilst pregnant, ii) If a vaccine against coronavirus (COVID-19) becomes available, I would get vaccinated whilst not pregnant and iii) If a vaccine against coronavirus (COVID-19) becomes available, I would vaccinate my baby.’ Responses were scored on a Likert scale: ‘Yes definitely’, ‘Unsure but leaning towards yes’, ‘Unsure but leaning towards no’, ‘No, definitely not’. This question was followed by a free-text box titled: ‘Feel free to add any additional comments here’.

This anonymous survey gathered optional demographic data including ethnicity, age, number of children, country of residence, region of residence in England, ethnicity, parity, income, pregnancy status, gestation at survey completion for those who were pregnant and date of delivery for those who had already had their babies. At the end of the online survey, participants were invited to take part in a follow-up interview by leaving their contact details; they were informed that by leaving their details, their responses would no longer be anonymous.

### Survey analysis

Responses were scored on a Likert scale coded as follows: 1) ‘Yes definitely’, 2) ‘Unsure but leaning towards yes’, 3) ‘Unsure but leaning towards no’, 4) ‘No, definitely not’. Acceptability of a COVID-19 vaccine for women when pregnant, when not pregnant and for their child was compared in all women surveyed by Pearson’s chi-square followed by analysis of each cell’s contribution to the chi squared statistic. Ordered logistic regressions of the Likert responses were conducted to determine the demographic factors associated with maternal acceptability of the COVID-19 vaccine for themselves whilst pregnant and not pregnant, and for their child. Ethnicity, age, country of residence, region of residence (for residents in England) and household income, were analysed separately and then in multivariate models to determine predictors of acceptability across the UK and within England. Odds ratios over 1 represent an increased likelihood of women’s responses moving from ‘yes, definitely’ towards ‘definitely not, and odds ratios of less than 1 represent a movement in the opposite direction. Some groups were combined as follows. 1) The <20y group was combined with the next age bracket to form < 25y age bracket. The ten income groups were combined pair-wise to form five groups. To enable multiparametric analysis, ethnicity was dichotomised into ‘White’ (i.e., White British, White Irish and White Other participants) and ‘Ethnic minorities’ (i.e., Black, Asian, Chinese, Mixed ethnicities or Other ethnicity). A *p* value of less than 0.05 was considered statistically significant. Sankey diagrams were created using SankeyMATIC.

### Semi-structured interviews

Participants who had left their contact details at the end of the online survey were contacted by SBa. Participants were purposively selected to prioritise women who; 1) were from ethnic minority backgrounds, due to lower representation among women surveyed; 2) were pregnant at the time of survey completion, due to their proximity to their pregnancy experience compared to those that had already had their babies at the time of survey completion; 3) had not completed the open text survey responses. Informed consent was obtained by telephone or e-mail, depending on participant preference. The participant information sheet and consent form were provided by e-mail (see supplementary material A). Interviews lasted approximately 30 min and were conducted over the telephone and/or using Microsoft Teams and were recorded with permission of the participant. Interviews were conducted by SBa and HS using a topic guide, which was developed based on the questionnaire (see supplementary material B). The data present their views on accepting the COVID-19 vaccine when pregnant, not pregnant and for their children. The interviews took place between 7th-16th December 2020.

### Qualitative analysis

Free-text responses following the survey questions on COVID-19 vaccine acceptance were analysed thematically by SBa using the stages outlined by Braun and Clarke: data familiarisation, coding and theme identification and refinement [23]. To enhance the rigour of the analysis, coding approaches and subsequent theme generation and refinement was discussed between HS, SBa, SBe and BH. Interviews were transcribed verbatim and analysed thematically following a similar approach as the free-text survey responses, initially by SBa and then with agreement by SBa, SBe HS and BH.

### Ethical approval

This study was approved by Imperial College Research Ethics Committee (ICREC) (Ref: 20IC6188).

### Data statement

All of the data supporting this research publication is included in the manuscript and supplementary material.

## Results

### Demographics of surveyed women

**Fig. 1:**
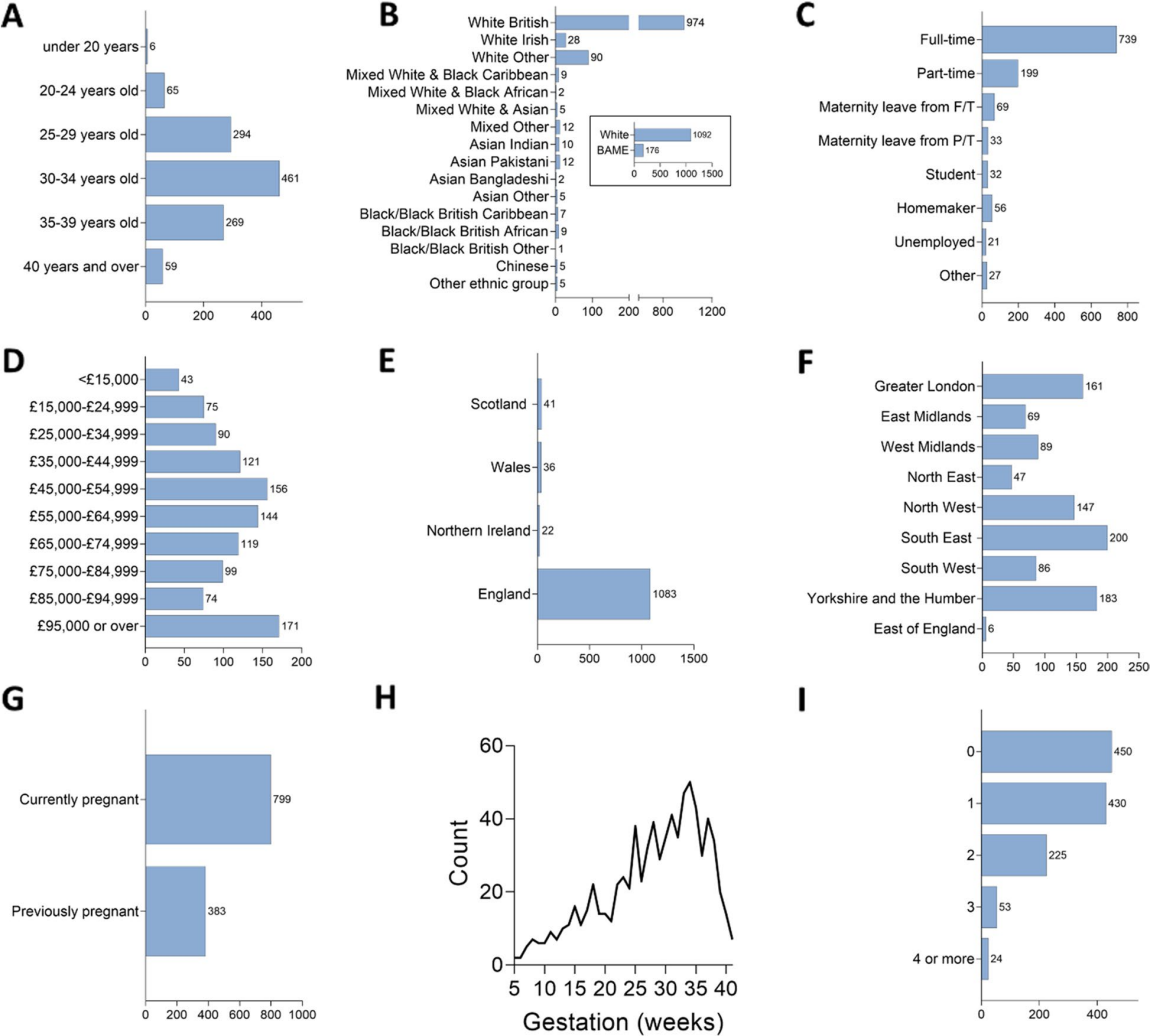
Demographics of Survey Respondents. Self-reported demographics of the survey respondents at the time of survey completion. These questions were optional. **A** Age (years); **B** Ethnicity; **C** Employment status; **D** Annual household income (£); **E** Country of residence; **F** Region of residence for English residents (*n*=988); **G** Pregnancy status; **H** Gestational age of pregnancy (weeks) for those currently pregnant; **I** Number of children

There were 1526 responses to the survey in total. 122 responses were excluded because they were test responses or incomplete responses, leaving 1404 responses. Of these, there were 1181 answers to the questions regarding acceptability of COVID-19 vaccination. Demographic details, for those women that chose to provide these, are summarised in Fig. 1. The most common age group was 30-34 years (*n* = 461), and the majority of women were White British (*n* = 1092). Most women worked full-time (*n* = 739), and median household annual income was £45,999-54,999. The majority of women were from England (*n* = 1083) though a higher proportion of women were from London and the South-East regions of England (see Fig. 1F). At the time of survey completion 68% of women were pregnant, whilst the remaining 32% had given birth. Those who were pregnant ranged from between 5 to 41 weeks’ gestation, with 34 weeks being the most frequent gestation (*n* = 50 - see Fig. 1H). Most women responding either had no other children (*n* = 450) or one child (*n* = 430).

### Acceptability of a future COVID-19 vaccine

**Fig. 2:**
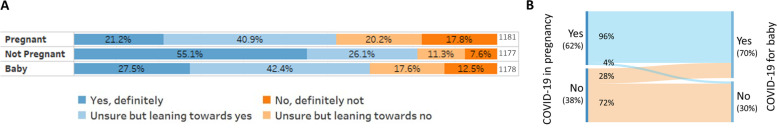
Women’s acceptability of a future COVID-19 vaccine during pregnancy, after pregnancy and for their baby. Survey respondents were asked how much they agreed or disagreed about a future vaccine to protect against COVID-19 for delivery whilst they were Pregnant, whilst Not Pregnant or for their Baby. Responses were scored on a Likert scale (see key). **A** All responses to the survey question (*n*=1177-1181); **B** Sankey plot of all respondents showing linkage between their acceptance of a future COVID-19 vaccine during pregnancy and their acceptance of the same vaccine for their babies (*n*=178)

The majority (81.2%) of women surveyed reported that they would ‘definitely’ accept (55.1%), or were ‘leaning towards’ (26.1%) accepting a future COVID-19 vaccine for themselves when not pregnant (Fig. 2A). Most women (62.1%) also reported that they would definitely accept, or were leaning towards accepting a future COVID-19 vaccine for themselves whilst pregnant. Acceptance of vaccination during pregnancy was significantly lower than acceptance outside of pregnancy (*p* < 0.005; Fig. 2A). Analysis of the contribution to this chi-square result showed that this was mainly driven by a difference in selection of ‘no, definitely not’, with 2.3 times more women expressing this perspective regarding vaccination in pregnancy (17.8%), compared to 7.6% regarding vaccination whilst not pregnant.

Most (69.9%) women surveyed reported that they would definitely accept (27.5%), or were leaning towards (42.4%) accepting a future COVID-19 vaccine for their babies (Fig. 2A). This was significantly lower than their acceptability of COVID-19 vaccination for themselves (*p* < 0.005; Fig. 2A). Almost all (96%) of women who reported they were likely to accept COVID-19 vaccination for themselves during pregnancy, also reported they were likely to accept the vaccine for their babies, compared to only 28% of those who reported they were likely to reject the vaccine for themselves (Fig. 2B).

### Acceptability of future COVID-19 vaccine depends on current pregnancy status

**Fig. 3:**
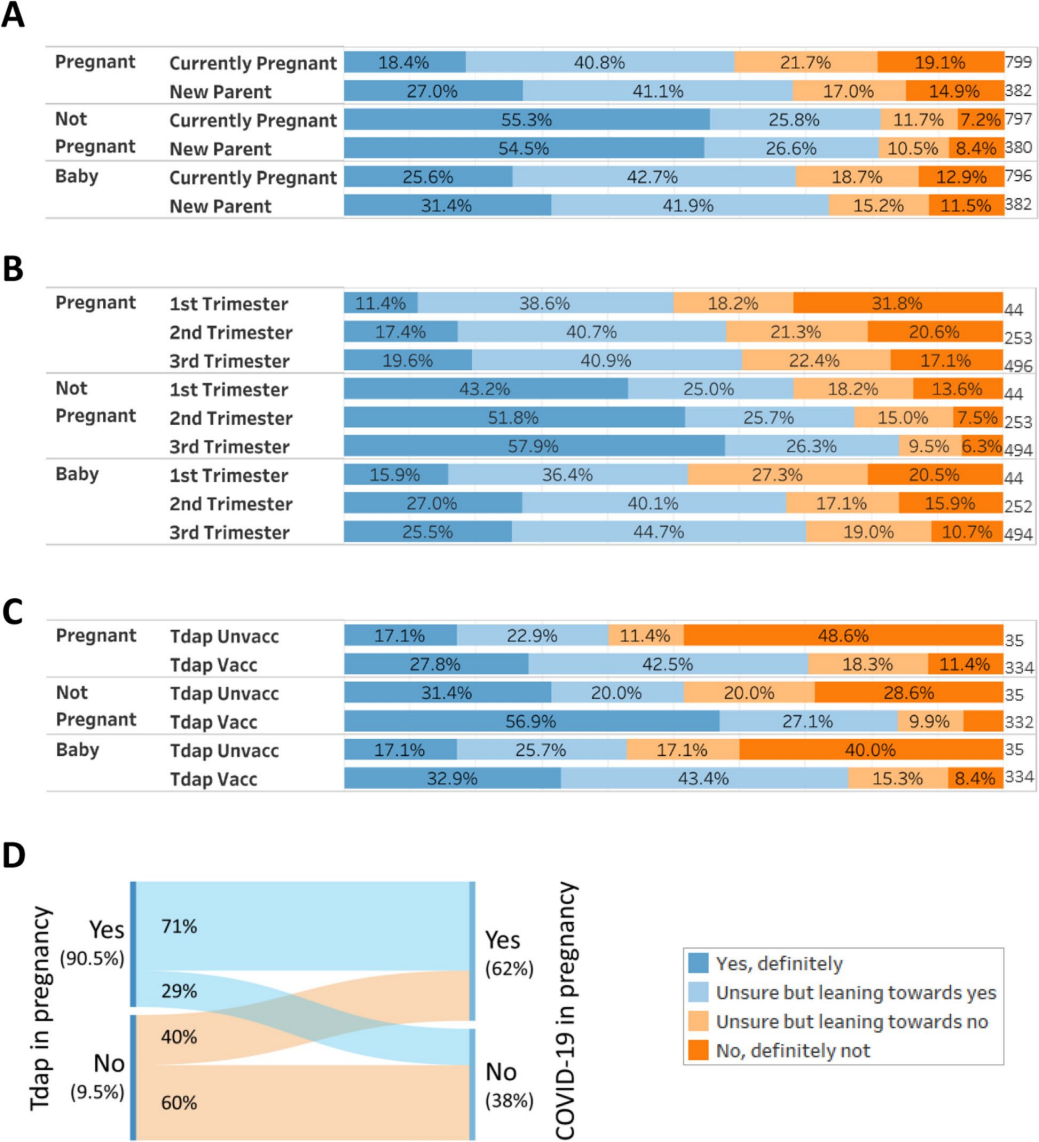
Women’s acceptability of a future COVID-19 vaccine during pregnancy, after pregnancy and for their baby, by pregnancy status. Survey respondents were asked how much they agreed or disagreed about a future vaccine to protect against COVID-19 for delivery whilst they were Pregnant, whilst Not Pregnant or for their Baby. Responses were scored on a Likert scale (see key). **A** COVID-19 vaccine acceptance split by whether women were pregnant at the time of the survey (*n*=796-799), or were new mothers (*n*=382); **B** COVID-19 vaccine acceptance split by gestation at time of survey completion; **C** COVID-19 vaccine acceptance in new mothers (*n*=382) split by whether they had been vaccinated against pertussis (Tdap vacc) or not (Tdap unvacc) during their last pregnancy; **D** Sankey plot of new mothers (*n*=382) showing linkage between Tdap vaccination status in their last pregnancy and their acceptance of a future COVID-19 vaccine in pregnancy

Women who were pregnant when they completed the survey were more likely to reject the idea of receiving a COVID-19 vaccine during pregnancy compared to those who were no longer pregnant when they completed the survey (*p* < 0.005; Fig. 3A). Among pregnant women, 59.2% said they would definitely or were leaning towards accepting a future vaccine during pregnancy, compared to 68.1% of women who were no longer pregnant. Women who had already had their baby were also more likely to accept the idea of receiving a COVID-19 vaccine for their babies, compared to those who were still pregnant (*p* < 0.005, Fig. 3A).

Of women who were pregnant at time of survey completion, those in the third trimester of pregnancy were more likely to accept the idea of a future COVID-19 vaccine for their baby compared to women in the first trimester (*p* = 0.025; Fig. 3B). They also had a non-significant higher likelihood of accepting a future COVID-19 vaccine during pregnancy (*p* = 0.057).

### Acceptability of future COVID-19 vaccine depends on previous pregnancy vaccine uptake

Of women who had delivered their babies, those who had not been vaccinated against pertussis in pregnancy were around four times (*p* value < 0.0005) more likely to reject the COVID-19 vaccine during pregnancy, outside of pregnancy and for their baby (Fig. 3C). Only 40% of unvaccinated women reported they were likely to accept a future COVID-19 vaccine, compared to 71% of pertussis-vaccinated women (Fig. 3D).

### Demographic factors are associated with COVID-19 vaccine acceptability

#### Ethnicity

**Fig. 4:**
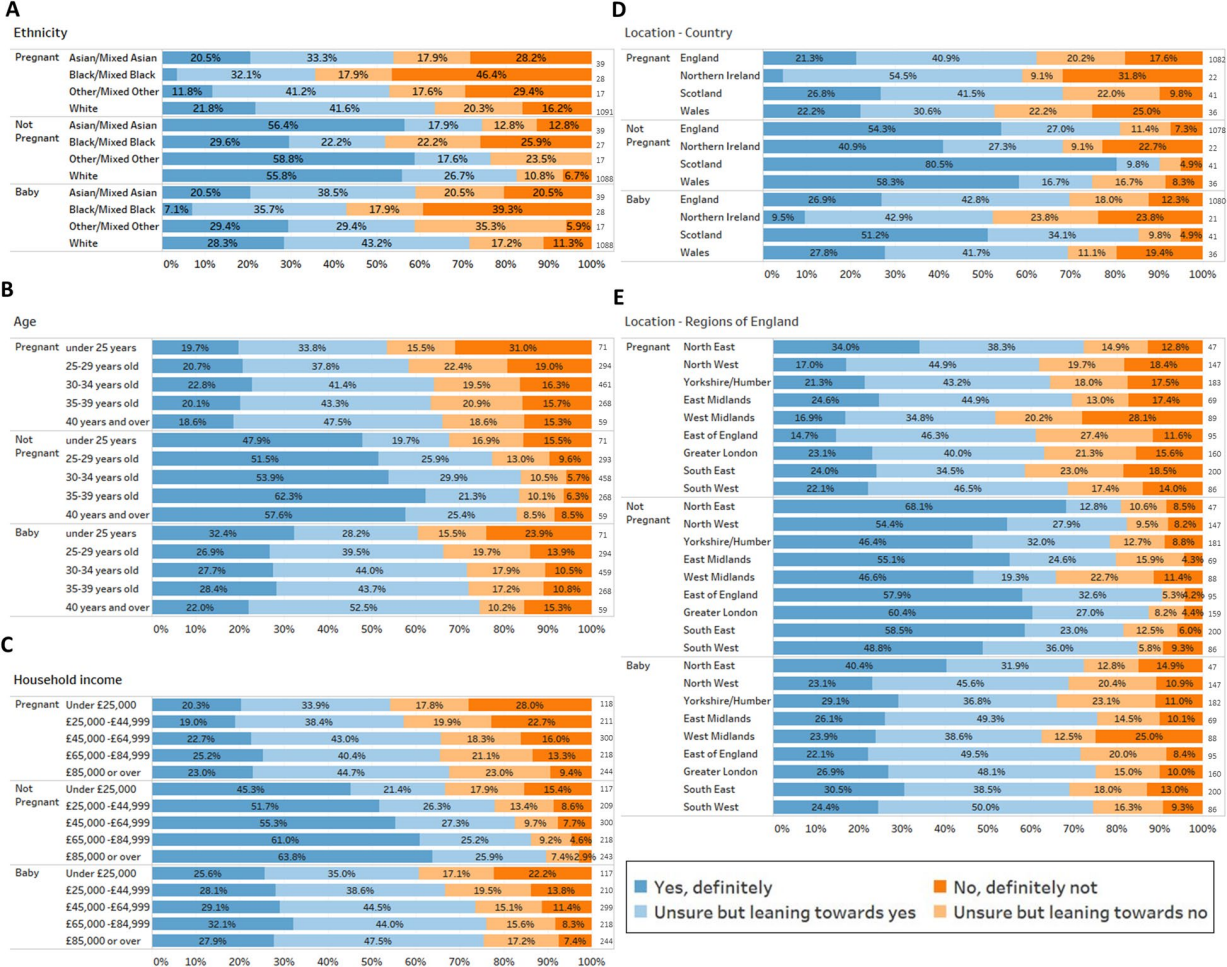
Women’s acceptability of a future COVID-19 vaccine during pregnancy, after pregnancy and for their baby split by respondent demographics. Survey respondents were asked how much they agreed or disagreed about a future vaccine to protect against COVID-19 for delivery whilst they were Pregnant, whilst Not Pregnant or for their Baby. Responses were scored on a Likert scale (see key). Responses are separated by self-reported demographics. These questions were optional, so the number of responses vary. The number in each group is shown to the right of each bar. **A** COVID-19 vaccine acceptance split by ethnicity. **B** COVID-19 vaccine acceptance split by age; **C** COVID-19 vaccine acceptance split by household income; **D** COVID-19 vaccine acceptance split by country of residence; E COVID-19 vaccine acceptance split by region of residence for English residents

Compared to women of white ethnicities, women from Black and Mixed Black ethnicity groups (Black-British African, Black-British Caribbean, Black-other, Mixed White-Black Caribbean, and Mixed White-Black African) were more likely to reject a future COVID-19 vaccine when pregnant, not pregnant or for their baby (*p* < 0.005, Fig. 4A, Supplementary Table 1). Among all white ethnicities 16.2% reported that they would definitely not accept a future COVID-19 vaccine when pregnant compared to 46.4% of Black and Black Mixed ethnicity women (Fig. 4A). Similarly, 21.8% of White women answered that they would definitely accept a future COVID-19 vaccine compared to 3.6% of Black and Mixed Black ethnicity women (Fig. 4A). The effect of ethnicity remained significant in multivariate analysis (Table 1).

**Table 1:** Multivariate analysis of predictors of COVID-19 vaccine acceptance

Variable									
	Pregnant			Non-pregnant			Baby		
	OR	95% CI	*P* value	OR	95% CI	*P* value	OR	95% CI	*P* value
Ethnicity									
White#	–	–	**–**	–	–	**–**	–	–	**–**
Minority ethnicity	2.20	1.45, 3.33	**< 0.005**	1.86	1.21, 2.87	**0.005**	2.17	1.43, 3.28	**< 0.005**
Age									
Under 25y	1.13	0.69, 1.88	0.614	1.10	0.66, 1.84	0.706	0.89	0.53, 1.49	0.668
25 – 29y	1.11	0.84, 1.45	0.473	1.07	0.80, 1.43	0.636	1.05	0.80, 1.39	0.712
30 – 34 y #	–	–	–	–	–	–	–	–	–
35 – 39 y	1.03	0.78, 1.36	0.845	0.75	0.55, 1.03	0.075	0.92	0.69, 1.22	0.573
Over 39y	0.91	0.56, 1.49	0.713	0.82	0.48, 1.42	0.484	0.99	0.60, 1.64	0.976
Country									
England#	–	–	–	–	–	**–**	–	–	**–**
Scotland	0.75	0.42, 1.33	0.321	0.31	0.14, 0.68	**0.004**	0.37	0.20, 0.68	**0.001**
Wales	1.25	0.66, 2.36	0.500	0.87	0.44, 1.74	0.436	1.02	0.53, 1.94	0.957
Northern Ireland	1.73	0.80, 3.74	0.163	2.02	0.89, 4.58	0.890	2.22	1.01, 4.85	**0.046**
Income									
Under £24,999	1.98	1.36, 2.89	**< 0.005**	2.59	1.73, 3.86	**< 0.005**	2.18	1.50, 3.18	**< 0.005**
£25,000-£44,999	1.53	1.08, 2.18	**0.017**	1.66	1.14, 2.43	**0.009**	1.28	0.90, 1.82	0.174
£45,000-£64,999	1.15	0.84, 1.58	0.384	1.42	1.00, 2.01	**0.048**	1.09	0.79, 1.50	0.598
£65,000-£84,999	1.06	0.76, 1.48	0.736	1.14	0.79, 1.66	0.484	0.94	0.67, 1.31	0.701
Over £85,000#	–	–	–	–	–	–	–	–	–

*OR* Ordinal odds ratio. An OR above 1 indicates a higher likelihood of women giving responses moving from ‘definitely yes’ towards ‘definitely no’ on the Likert scale. *95% CI* 95% confidence interval. # indicates the comparator group in the analysis. Ethnicity Groups: White: White-British, White-Irish, White-Other; Minority ethnicity: Black-British African, Black-British Caribbean and Black-Other, Asian Indian, Asian-Pakistani, Asian-Bangladeshi, Asian-Other and Chinese, Mixed White-Black Caribbean, Mixed White-Black African, Mixed White-Asian, Mixed White/Other and Other ethnicity

#### Age

In univariate analysis, women aged under 25 years were more likely to reject a future COVID-19 vaccine whether pregnant or not pregnant, compared to the middle age bracket of 30-34 (*p* < 0.05 –Fig. 4 B and supplementary Table 1) but were equally accepting of a future COVID-19 vaccine for their baby (*p* > 0.05). In the multivariate analysis, which also considered ethnicity, location and income, the relationship between younger age and being more likely to reject a future COVID-19 vaccine was no longer significant (Table 1).

#### Income

Compared to the highest income bracket, women with lower annual household incomes were more likely to reject a COVID-19 vaccine for themselves and for their babies (Fig. 4C and Supplementary Table 1). Women with household incomes less than £25,000 were 3 times more likely to say definitely no to a COVID-19 vaccine in pregnancy (28%) compared to the highest income bracket (>£80,000; 9.4%). In multivariate analysis, which considered ethnicity, location and age, lower income remained significantly associated with reduced acceptance (Table 1).

#### Geographical location – country of the United Kingdom

Women from Scotland were more likely to accept a future COVID-19 vaccine for themselves whilst not pregnant and for their baby compared to women from England (*p* < 0.05; Fig. 4D, Supplemental Table 1). Almost twice the number of Scottish women responded ‘definitely yes’ (51.2%) to a vaccine for their baby compared to English women (26.9%). These differences remained once ethnicity, income and age were considered (Table 1). Similarly, women from Northern Ireland were more likely to reject a future COVID-19 vaccine for their babies compared to women from England (*p* = 0.032; Fig. 4D) which also remained significant in multivariate analysis (see below).

#### Geographical location – region of England

In univariate analysis, women located in the West Midlands were more likely to reject a future COVID-19 vaccine for themselves when pregnant (*p* = 0.027), not pregnant (*p* = 0.002) and for their babies (*p* = 0.042; Fig. 4E) compared to women from Greater London. Women from Yorkshire were also more likely to reject a future COVID-19 vaccine when not pregnant (*p* = 0.006; Fig. 4E). These regional differences were lost once ethnicity, income and age were considered (Table 1). Stepwise inclusion of variables indicated that the lower acceptance in the West Midlands was accounted for by income rather than ethnicity (data not shown).

### Multivariate analysis of demographic factors associated with COVID-19 vaccine acceptability

We performed two multivariate logistic regressions-one for all UK women (Table 1) and one for women from England (Supplementary Table 2), considering ethnicity, age, household income and location. Across the UK, predictors of lower acceptance of a future COVID-19 vaccination when pregnant were being from an ethnic minority (*p* < 0.005), and having a household income lower than £45,000 (*p* = < 0.005 for <£25,000; *p* = 0.017 for £25,000-44,999). For acceptance of a future COVID-19 vaccine when not pregnant, being from an ethnic minority (*p* = 0.005) and having a household income below £65,000 (*p* = < 0.0005 for <£25,000; *p* = 0.009 £25,000-44,999; *p* = 0.048 for £45,000-64,999) were also associated with lower acceptance. In addition, being a resident of Scotland was independently associated with higher acceptance (*p* = 0.004). Predictors of lower acceptance of a future COVID-19 vaccine for women’s babies were being from an ethnic minority (*p* = < 0.005) and having a household income below £25,000 (*p* = < 0.005). Being a resident of Scotland was also associated with higher acceptance of a future COVID-19 vaccine for babies (*p* = 0.001) while women from Northern Ireland were more likely to reject a future COVID-19 vaccine for their baby (*p* = 0.046).

Within England, following inclusion of other variables, the association between women living in the West Midlands being more likely to reject a COVID-19 vaccine became non-significant (*p* = 0.058). Once ethnicity, location and income were considered, age was no longer a predictor of vaccine acceptance for any scenario nor living in any region of England. See Table 1 and supplementary Table 2.

### Qualitative analysis reveals barriers and drivers of acceptance of novel pregnancy vaccines during a pandemic

Of the 1181 responses to the COVID-19 vaccine acceptance question in the survey, 19.7% (*n* = 233) left a response in the subsequent free-text box. The number of responses to each theme by survey respondent is shown in Table 2 including how often themes were mentioned by the women surveyed. Semi-structured interviews took place with 10 women who were from 6 different regions of England, the most common area being London where 4 of the women lived. The women interviewed were aged between 25 and 40 years and further demographics are displayed in Table 3.

**Table 2:** Free-text survey interview responses organised into themes

Theme	Women N, (%)
Concern about the safety of COVID-19 vaccines	141 (61)
Not enough data available	107 (46)
Speed of vaccine development	40 (17)
Possible longer term side effects	45 (19)
Trust in vaccines and the wider health system	115 (49)
Positive towards COVID-19 vaccines	41 (18)
Trust in vaccination	34 (15)
Trust in NHS advice	13 (6)
Mistrust in governmental advice and pharmaceutical companies	27 (12)

**Table 3:** Interviewee characteristics

No	Ethnicity	Parity	Tdap	Would accept COVID-19 vaccine-		
				-when pregnant	-when not pregnant	-for baby
1	Chinese	3	Yes	No	No	No
2	White Asian	1	Yes	Yes	Yes	Yes
3	British Pakistani	1	No	No	Yes	No
4	British Arabic	1	Yes	No	Yes	No
5	Black-African	1	Yes	No	No	No
6	White British	2	Yes	No	Yes	No
7	White British	4	Yes	Yes	Yes	Yes
8	White British	2	Yes	No	Yes	No
9	White British	2	Yes	Yes	Yes	Yes
10	White British	1	Yes	No	Yes	No

Thematic analysis of the freetext survey responses and interviews found that safety concerns (61% women surveyed who left freetext answers) were the most common reason women gave for influencing whether they would accept a COVID-19 vaccine during pregnancy. Safety concerns were linked to concerns about lack of safety data (46% women surveyed who left freetext answers) and worries around the speed of vaccine development (17% women surveyed who left freetext answers). Women also commonly (49% women surveyed who left freetext answers) mentioned trust or mistrust in wider vaccination and the health system as reasons for declining or accepting future COVID-19 vaccines. Thematic analysis of both the survey responses and interviews are outlined below.

#### Concern about the safety of COVID-19 vaccines

In free-text responses and the interviews, many women expressed safety concerns, particularly related to feeling that there was ‘not enough data available on the COVID-19 vaccines’ for either them or their babies to be vaccinated – a theme also linked to the speed of development of the COVID-19 vaccine. Women expressed that there was “Not enough evidence to say it would work, no evidence to say it doesn’t cause side effects.”(survey participant #S1212) or that they would like “to read more evidence/studies and science on vaccinations against Covid before considering my baby having it or myself whilst pregnant.” (survey participant #S1275). Women particularly wanted data on the safety of the vaccine on foetuses and whilst breastfeeding: “I’d want reassurance that there was absolute confidence that there were no harmful long-term effects on my baby or no chance of it causing any harm in utero’ (survey participant #S1228) and “I would also want more information on how it would affect my baby if I was breastfeeding, and I received it.” (survey participant #S1340). One interviewee described that “once it’s been declared safe for me to have it, I would go ahead and get it done”(Interviewee 09).

Another common safety concern expressed by women was the ‘speed of vaccine development’ of COVID-19 vaccines and therefore the ‘newness’ of any future COVID-19 vaccines. For example, one respondent reported: “I would be very cautious over a new vaccine that has been created in such short space of time when usually they take years.” (survey participant # S746). Whilst another respondent stated: “No one in their right mind would accept a vaccine that’s been rushed’ (survey participant #S1497). There was a feeling that with time after a COVID-19 vaccine had been widely used that this would increase acceptability amongst women: “My worry is that it hasn’t had long enough to understand any longer-term effects of the vaccine yet.” (survey participant #S1426). Another respondent said: “Vaccines take years to perfect, there is no way I am vaccinating either my baby or myself with a vaccine that has only just been found!” (survey participant #S1236). One interviewee said: “Because it’s really new, there’s not a lot of, um, like there’s not a lot of research into it yet. I know they’ve just taken it out and it’s like a short period of time otherwise vaccines take a good few years before they come out. So, I just think the timeframe and things.” (Interviewee 03).

Many comments reflected worries about the ‘possible long-term side effects of a Covid -19 vaccines’ not having been studied: “No long-term side effects or risks are known about any vaccine for coronavirus therefore I wouldn’t be comfortable vaccinating myself or my children.” (survey participant #S1499). This theme was commonly also related to wanting more evidence on safety such as for example: ‘I have to see what might be the long-term effects because now we don’t know about the long-term effects about the vaccine. Maybe it’s now it’s effective, right now, but no one knows what can happen with the people that get the vaccine now.” (Interviewee 05). The concerns about the side effects were also related to the speed of development: “I feel vaccines might be rushed without thorough long-term side-effect testing. So would want to wait for evidence they are 100% safe.” (survey participant #S1425).

#### Trust in vaccines and the wider health system

Women who expressed views that were ‘positive responses towards COVID-19 vaccines’ often mentioned this as part of a wider trust and confidence in vaccines and the health system: “I strongly believe in vaccination. I trust the UK’s method in developing a COVID vaccine and it’s safety.” (survey participant #S1457) and “I would trust my GP and midwives if they recommended it.” (survey participant #S1247).

Women therefore reported being confident and having ‘trust in vaccination’ in general and therefore being willing to accept the COVID-19 vaccine if it was recommended or deemed safe: “if told safe by researchers/GP, would get vaccinated in pregnancy for Covid 19 definitely.”(survey participant #S1317).

‘Trust in NHS advice’ was also expressed as a reason for accepting a future COVID-19 vaccine “I also understand that vaccinations for pregnant women and young babies would not be offered on the NHS if they weren’t safe. So, if they were being offered on the NHS then yes, I would have them.” (survey participant #S1529).

Concerns about the speed of the development of the vaccine in the context of the global pandemic also related to ‘mistrust in government’ regarding the handling of the COVID-19 pandemic and also ‘mistrust in wider pharmaceutical industry’. For example: **“**I feel it’s so early in understanding the virus to be vaccinated, all of the science has been confusing and changes far too often and doesn’t go far enough to answer why BAME communities are most at risk. I’m not comfortable with the advice being given and the government’s ability to be truthful so I will not be getting a vaccination and I would not want to put my baby at risk either” (survey participant #S1199 Q55).

Women also expressed mistrust about a COVID-19 vaccine for example: “I would not trust a vaccine against Covid so I would never get it or my baby. I feel it would be very dangerous to our health.” (survey participant #S1270). These sentiments were often expressed again as mistrust in vaccine manufacturing in general for example: “I feel very sceptical and paranoid about whether I was to get vaccinated if a COVID vaccine was available. This is due to scientists disagreeing with each other and the contradictions presented by vaccine developers” (survey participant #S1368).

#### Additional themes identified in interviews

Some additional themes not identified in the free-text responses emerged from the interviews. Interviewees commonly acknowledging ‘that children are not at increased risk from coronavirus’ which links to the lower likelihood of acceptance of infant vaccination seen in our quantitative analysis (Fig. 2A). For example: “children are less likely to have corona than maybe grown-ups, so I don’t think I will get my baby the vaccine.” (Interviewee 05). Interviewees also acknowledged that the COVID-19 vaccine offered a route to resuming normal life: “I feel, you know, they’ve passed it, it’s gone through vigorous testing. Yes, it may have been quite quick, but it needed to be quick obviously. I think the more people that are vaccinated, the quicker we may get back to some form of normality, it may not go back to what it was before, but I definitely think it’s a good thing”(Interviewee 06).

## Discussion

COVID-19 vaccines have been offered to all pregnant women in the United Kingdom since April 2021 [1]. Despite this the UK Obstetric Surveillance System has reported that 98% of pregnant women admitted to hospital with COVID-19 between February and September 2021 were unvaccinated [21]. Furthermore, 17% of the total critically ill patients in English hospitals on Extra Corporeal Membrane Oxygenation (ECMO) therapy were pregnant women [24]. Our survey of 1181 UK women explored COVID-19 vaccine attitudes for pregnant women during the first wave of the COVID-19 pandemic.

We found that the majority of women would accept or were leaning towards accepting a COVID-19 vaccine. Vaccine acceptability was highest when women were not pregnant, with over 8 in 10 of women answering they would likely accept COVID-19 vaccination. A significantly lower proportion of 6 in 10 women would likely accept a COVID-19 vaccine when pregnant. This supports previous findings that women are less likely to accept COVID-19 vaccination during pregnancy [25, 26]. Our findings of most pregnant women accepting a future COVID-19 vaccine contrast to a cohort study at one hospital in London that found only 28.5% of eligible pregnant women who gave birth between March 1 and July 4 2021 were vaccinated against COVID-19 [27]. Vaccine uptake among pregnant could have since increased in the UK, however understanding the drivers and barriers affected COVID-19 vaccine acceptance given their higher risk of hospitalisation remains timely and relevant.

Pregnant women in the UK have been offered vaccination against pertussis and seasonal influenza for a number of years. We found that women who had not been vaccinated against pertussis in pregnancy were four times more likely to also reject the COVID-19 vaccine during pregnancy. Declining seasonal influenza vaccine has also been associated with non-acceptance of the COVID-19 vaccine in pregnancy [28]. Uptake of existing maternal vaccines can be affected by both access and vaccine confidence [29] and in the UK women living in poorer areas or belonging to a minority ethnicity group are less likely to be vaccinated in pregnancy [19, 30, 31]. Ethnic minority children in the UK are also less likely to be vaccinated with their routine childhood vaccines [30, 32, 33]. Mirroring this, our findings also suggest that women belonging to an ethnic minority group were twice as likely to reject a COVID-19 vaccine for themselves when pregnant, not pregnant and for their babies compared to women from white ethnicity groups. In the London hospital cohort lower COVID-19 vaccine uptake among pregnant women of non-white ethnicity was also reported [27]. This is consistent with other UK surveys assessing COVID-19 vaccine acceptability for non-pregnant adults, which also found that ethnic minorities are less likely to be vaccinated [14, 16, 17]. A survey in the United States also reported lower COVID-19 vaccine acceptance among Black Afro-Caribbean and Hispanic American pregnant women compared to White women [28].

We found that women with lower annual household income were more likely to reject COVID-19 vaccination for their babies, and for themselves (when pregnant and not pregnant). Our findings suggest that the regional variation observed within England, or uptake in lower age groups is driven by lower income and ethnicity. Again this is supported by the hospital cohort study in London of women delivering between February and July 2021 that found lower COVID-19 vaccine uptake among lower income women [27]. Lower income can intersect with lower education levels [34] and vaccine hesitancy towards COVID-19 vaccines has been found to correlate with lower education levels [35]. Therefore, tailored, accessible information to overcome hesitancy among different population groups and ethnic minorities is needed in England [34, 36]. Women living in Scotland were more accepting of COVID-19 vaccination, independent of income and ethnicity, with 5 of 10 women saying they would definitely accept vaccination for their baby, compared to only 1 in 10 Northern Irish women.

Qualitative analysis of both the survey and the semi-structured interviews found that some women expressed concerns about COVID-19 vaccine development, as they perceived it as having been rushed and they wanted more information on safety and side-effects. Previous interviews with pregnant women in England in April and May 2020 found that pregnant women perceived the risk of COVID-19 vaccines as potentially greater than the risk from COVID-19 itself [37]. It would be beneficial to conduct a repeat survey now that there is more publicly-available data from large COVID-19 vaccine trials and COVID-19 vaccines are available to pregnant women. Our thematic analysis identified that women expressed confidence in accepting COVID-19 vaccines if they trusted vaccination in general, and/or if they thought they would be recommended by the NHS during pregnancy. This supports our finding that pertussis vaccination uptake in pregnancy predicted women’s acceptance of future COVID-19 vaccination. Some women expressed feelings of mistrust in COVID-19 vaccines, wider vaccination programmes and health system advice, which are areas that should be prioritised for public health messages.

The majority of women we surveyed would either accept or were leaning towards accepting a COVID-19 vaccine for their baby, although this was also significantly lower than vaccine acceptance for themselves. Our findings therefore support previous research relating pregnancy vaccine attitudes to parental vaccine decisions for children [20]. We found that women not vaccinated against pertussis in pregnancy were also four times to reject COVID-19 vaccination for their baby. Thus, improving vaccine information delivery in pregnancy may also improve subsequent childhood vaccine acceptance and indeed wider COVID-19 vaccine acceptance. Promoting vaccination in pregnancy [29] potentially therefore offers an opportunity to promote vaccination along the life course.

Our findings add weight to the calls to involve pregnant women in vaccine trials earlier, in order for vaccine safety data to be available so that pregnant women to make informed vaccine decisions [7, 38, 39]. Free-text responses and interview responses both showed that the main concern about COVID-19 vaccines was around safety. Lower acceptance of vaccination in pregnancy suggests that women are understandably more cautious about receiving a new vaccine whilst pregnant and they want safety information and data that is directly related to pregnancy. Thus, the earlier availability of vaccine safety data relating to pregnancy, and its communication, is vital. Since our survey and interviews were carried out there has been misinformation around COVID-19 vaccines and women of child-bearing age – including inaccurate rumours around the COVID-19 vaccine impacting on fertility meaning accurate safety communication to all women is needed [40–42].

### Strengths and limitations

The main strength of this study was the use of multiple methods – the qualitative analysis of the survey and interviews enabled factors behind the quantitative findings to be explored in more detail. The response rate with over 1000 responses from women who had been pregnant during the first peak of the COVID-19 pandemic in the UK was excellent with nearly 1 in 5 women leaving freetext responses to the COVID-19 vaccine acceptability questions.

The survey included women from across the United Kingdom, which enabled us to identify interesting differences in Scotland and Northern Ireland compared to England, however the majority of women were from England. Regionally, London and the South-East were overrepresented, however the survey included women with a range of ages and income levels and at different pregnancy gestations. Importantly, the survey captured women with a range of vaccine attitudes and women who had both been vaccinated with pertussis vaccine in pregnancy and those who had not. Although we were able to detect a significantly lower uptake in some ethnic minority groups (Black ethnicities including Black-British African, Black-British Caribbean, Black-other and Black Mixed ethnicities) we were underpowered to detect differences in other ethnic groups. Thus, to explore the role of ethnicity in multivariate analyses we had to combine ethnic groups and ethnicity was dichotomised into White ethnicities and all other ethnic minorities.

The strength of this survey was that it took place in the middle of the pandemic, at a time of great uncertainty and upheaval for both patients and the healthcare system. However, this is also the main limitation of the survey, as when the survey was live between August and October 2020 no COVID-19 vaccine had yet been licensed. The survey may have found different results if it had taken place once the COVID-19 vaccines had been approved in the United Kingdom as studies have found that vaccine attitudes towards the COVID-19 vaccines have changed over time [43].

## Conclusion

Our findings support the need for clear accurate communication to reassure pregnant women about COVID-19 vaccine safety particularly given the number of pregnant women admitted to hospital with COVID-19. Monitoring COVID-19 vaccine uptake among different income and ethnicity groups is needed to ensure existing inequalities in vaccine uptake among pregnant women are not exacerbated. Targeted interventions to maximize COVID-19 vaccine uptake among pregnant women from ethnic minority communities and those living in deprived areas should be explored.

## Supplementary Information

**Additional file 1.**

## Data Availability

All of the data supporting this research publication is included in the manuscript and supplementary material.
